# Diabetic retinopathy classification for supervised machine learning algorithms

**DOI:** 10.1186/s40942-021-00352-2

**Published:** 2022-01-03

**Authors:** Luis Filipe Nakayama, Lucas Zago Ribeiro, Mariana Batista Gonçalves, Daniel A. Ferraz, Helen Nazareth Veloso dos Santos, Fernando Korn Malerbi, Paulo Henrique Morales, Mauricio Maia, Caio Vinicius Saito Regatieri, Rubens Belfort Mattos

**Affiliations:** 1 Physician, Department of Ophthalmology, Universidade Federal de São Paulo - EPM, Botucatu Street, 821, Vila Clementino, São Paulo, SP 04023-062 Brazil; 2 Instituto Paulista de Estudos e Pesquisas em Oftalmologia, IPEPO, Vision Institute, São Paulo, SP Brazil; 3 NIHR Biomedical Research Centre for Ophthalmology, Moorfield Eye Hospital, NHS Foundation Trust, and UCL Institute of Ophthalmology, London, UK

**Keywords:** Diabetic retinopathy classifications, Artificial intelligence, Datasets

## Abstract

**Background:**

Artificial intelligence and automated technology were first reported more than 70 years ago and nowadays provide unprecedented diagnostic accuracy, screening capacity, risk stratification, and workflow optimization.

Diabetic retinopathy is an important cause of preventable blindness worldwide, and artificial intelligence technology provides precocious diagnosis, monitoring, and guide treatment. High-quality exams are fundamental in supervised artificial intelligence algorithms, but the lack of ground truth standards in retinal exams datasets is a problem.

**Main body:**

In this article, ETDRS, NHS, ICDR, SDGS diabetic retinopathy grading, and manual annotation are described and compared in publicly available datasets. The various DR labeling systems generate a fundamental problem for AI datasets. Possible solutions are standardization of DR classification and direct retinal-finding identifications.

**Conclusion:**

Reliable labeling methods also need to be considered in datasets with more trustworthy labeling.

## Background

Computers executing automated functions were first described in 1950, with the first publication in 1943. Since then, Artificial Intelligence capacity has evolved into deep learning and neural networks, technologies that could simulate interconnected neurons and provide outputs after multiple information layers [1, 2].

Automated technology provides unprecedented diagnostic accuracy, screening capacity, risk stratification, and workflow optimization with accuracy equivalent to healthcare professionals [3] and more cost-effective diseases screening [4].

In Machine Learning, supervised learning is the most applied method in disease screening and classification algorithms, corroborating the importance of data labeling quality [5, 6].

Diabetic retinopathy (DR) is the leading cause of preventable blindness in working-age adults worldwide [7, 8], responsible for more than 24,000 annual cases of blindness [9] and the main focus in Ophthalmological AI screening algorithms [10]. There is an increased blindness risk in patients with chronic diabetes mellitus, especially those with poor clinical control [11].

Telemedicine and automated screening programs could diagnose, monitor, and guide treatment. Precocious diagnosis and therapy could avoid severe vision loss in 90% of cases, but only 60% of diabetic patients have recommended yearly examinations [12].

There are many Diabetic Retinopathy classifications applied in distinct countries and screening programs, with the International Council of Ophthalmology Diabetic Retinopathy (ICDR) classification as the most applied in open-access ophthalmological datasets [13].

High-quality retinal exams are fundamental in the development of AI algorithms, but also standards in labeling protocols, classifications, and quality control. This article describes and compares the most commonly diabetic retinopathy classifications, referencing criteria, and their applications in datasets.

## Main text

This study compared the most often-applied DR classification scales: Scottish Diabetic Retinopathy Grading [14], Early Treatment Diabetic Retinopathy Grading [15], International Clinic Diabetic Retinopathy [16], National Health Service Diabetic Retinopathy Classification grading [17], Modified Davis Retinopathy staging [18], and direct findings identification.

### The Early Treatment Diabetic Retinopathy Study

At an international consortium of ophthalmologists at Airlie House in 1968, internists and neurosurgeons standardized a diabetic retinopathy classification applied in the landmark Early Treatment Diabetic Retinopathy Study [15], designed to generate a more precise staging for DR and macular edema. The study screened for the presence of microaneurysms (MA), retinal hemorrhages, cotton-wool spots, intraretinal microvascular abnormalities (IRMA), venous beading, and neovessels in 35-mm photographs. The consortium provided standard photos of microaneurysms, hemorrhages, and neovessels.

The ETDRS defined microaneurysms as red spots of less than 125 microns in its longest dimension with well-delimited margins and defined hemorrhage as a red spot with irregular margins with more than 125 microns. Punctate lesions, blots, linear hemorrhages, and microaneurysms were classified as red spots when they were not distinguished in ETDRS charts [19].

ETDRS defined clinically significant macular edema as retinal edema seen in retinal stereo photographs at or within 500 microns of the center of the macula or hard exudates at or within 500 microns of the foveal center and retina thickening or retinal thickening larger than one disc diameter area within one disc diameter of the center of the macula. In 2006, Rudnisky compared modified ETDRS protocols with one or two fields and 16:1 JPEG images and showed good reproducibility compared to standard ETDRS stereoscopic photos [20]. (Table 1).

**Table 1:**
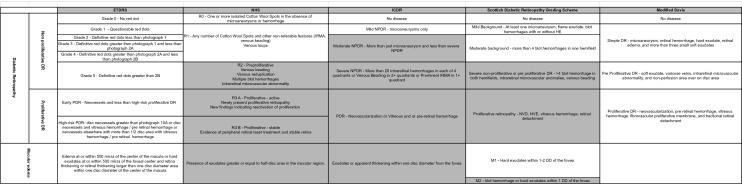
Comparison of ETDRS, NHS, ICDR, SDRGS, Modified Davis diabetic retinopathy scales

Immediate referable classifications are in grey color, when available criteria

### National Health Service diabetic retinopathy classification

The National Health Service (NHS) was a diabetic retinopathy classification system applied In England, Scotland, Wales, and Northern Ireland between 2002 and 2007. It applied an ETDRS modified diabetic retinopathy scale classified in four severity stages [17, 21]. This program evaluated and classified DR using macula-centered and optic disc-centered images [22]. The NHS screening program provided guidelines for grading and lesions classifications [23].

This DR classification considered macular exudates sign of macular edema because the images were non-stereoscopic; it also added a photocoagulation classification (Table 1).

### International Clinic Diabetic Retinopathy

The International Clinic Diabetic Retinopathy (ICDR) classification was published in 2003 after a consensus of 31 retina specialists, endocrinologists, and epidemiologists from 16 countries and sponsored by the American Academy of Ophthalmology [16]. The ICDR classified DR on a five-stage severity scale and classified diabetic macular edema as apparently absent or present. The classification was created to simplify the ETDR and Wisconsin Epidemiologic Study scale and make it more applicable in daily practice studies [16].

ICDR is applied in the EYEPACS dataset [24], Asian Pacific Tele-Ophthalmology Society dataset [25], Indian Diabetic Retinopathy Image Dataset [26], Messidor 1 and 2 datasets [27] (Table 1).

### The Scottish Diabetic Retinopathy Grading Scheme, 2004

In 2003, the National Scotland Eye Screening for Diabetic Retinopathy Program was created [28]. This grading system classified DR in all patients aged 12 years and older. Retinal digital photos were analyzed, and the re-screening period or ophthalmologist referral was established. The Scottish diabetic retinopathy grade (SDRG) is divided into four DR severities in a single fovea-centered image with at least two disc diameters temporal to the fovea and one disc diameter nasal to the disc [14] (Table 1).

### Modified Davis retinopathy staging

The ICDR score simplifies DR in three stages: simple diabetic retinopathy, pre-proliferative retinopathy, and proliferative retinopathy using 45-degree photographs of the posterior pole applied in the Jichi DR dataset [18] (Table 1).

### Direct findings identification

In AI datasets, findings such as microaneurysms, hemorrhages, hard exudates, and retinal detachment could be identified through direct identification. Applications such as SuperAnnotate [29], VGG Image annotation Tool [30], Supervise.ly [31], Labelbox [32], and Visual Object Tagging Tool [33] are available as labeling tools.

**Fig. 1:**
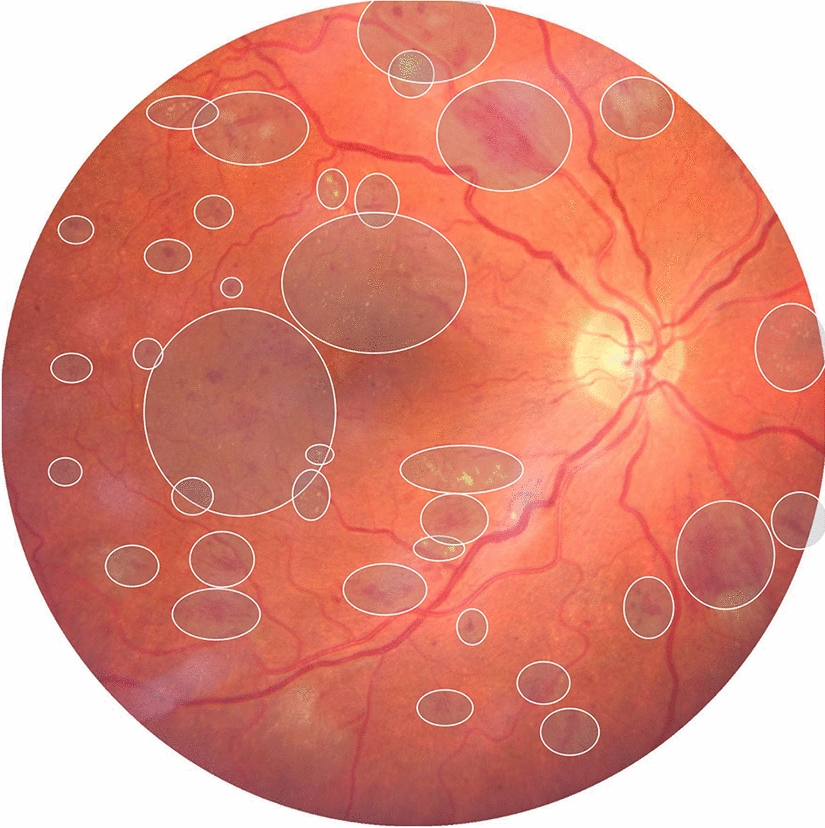
Direct retinal findings manual annotation example, in Labelbox software

In ODIR [34], DIARETDB 0 and 1 [35], DR 1 and 2 [36], E-Ophtha [37], and HEI-MED [38], retinal findings are manually annotated (Fig. 1).

### Referencing criteria comparison

The NHS, ICDR, and SDRGS establish referencing criteria. In NHS and SDRGS, the criteria are similar, with multiple retinal hemorrhages, intraretinal microvascular anomalies, or venous beading. In the ICDR, should be referenced patients with more than just microaneurysm, a criterion with greater sensitivity [14, 16, 17].

Considering macular edema, the NHS, SDRGS, and ICDR recommend referencing patients with exudates or apparent thickening in the macular area. The NHS recommends exudates distance within half-disc diameter from the fovea and ICDR and SDRGS within one disc diameter [14, 16, 17] (Table 1).

## Conclusions

Artificial intelligence and automated technology were first reported more than 70 years ago and nowadays provide unprecedented diagnostic accuracy, screening, risk stratification, and workflow optimization [3].

Reliable datasets are fundamental in supervised Machine Learning development; however, labeling process standardization, quality control, and homogenization remain challenging [39].

In diabetic retinopathy, there are distinct DR classifications, with different numbers of DR gradings and methods such as the Scottish Diabetic Retinopathy Grading [14], Early Treatment Diabetic Retinopathy Grading [15], ICDR [16], NHS Diabetic Retinopathy Classification grading [17], and Modified Davis Retinopathy staging [18] that are described in this review. Still, direct retinal findings annotation is valuable in neural networks training.

The Scottish Diabetic Retinopathy Grading is a valuable classification through retinal photographs due to a single macular centered retinal evaluation and is more sensitive for grading moderate and severe cases than ICDR classification.

When choosing the classification method applied in the dataset, the image field of view and the number of images must be considered. Classical ETDRS and ICDR classifications tend to underestimate DR classification in retinal photographic images due to limited image view areas compared to retinal fundus examinations.

The various DR labeling systems generate a fundamental problem for AI datasets, and it is fundamental to standardize DR grading in datasets to develop algorithms and ensure proper patient referral. Reliable labeling methods also need to be considered in datasets with more trustworthy labeling.

## Data Availability

Not applicable.

## Abbreviations

ETDRS: Early Treatment Diabetic Retinopathy Grading
NHS: National Health Service Diabetic Retinopathy Classification grading
ICDR: International Council of Ophthalmology Diabetic Retinopathy
SDRGS: Scottish Diabetic Retinopathy Grading
AI: Artificial intelligence
